# Polarization-Dependent SFG Spectroscopy of Near Ambient Pressure CO Adsorption on Pt(111) and Pd(111) Revisited

**DOI:** 10.1007/s11244-018-0949-7

**Published:** 2018-04-13

**Authors:** Xia Li, Matteo Roiaz, Verena Pramhaas, Christoph Rameshan, Günther Rupprechter

**Affiliations:** Institute of Materials Chemistry, TU Wien, 1060 Vienna, Austria

**Keywords:** Sum frequency generation, Carbon monoxide, Single crystals, Orientation, Model catalysis

## Abstract

Polarization-dependent sum frequency generation (SFG) vibrational spectroscopy was employed to examine CO overlayers on Pt(111) and Pd(111) single crystal surfaces at room temperature. Utilizing different polarization combinations (SSP and PPP) of the visible and SFG light allows to determine the molecular orientation (tilt angle) of interface molecules but the analysis of the measured $I_{\text{ppp}}/I_{\text{ssp}}$ is involved and requires a proper optical interface model. For CO/Pt(111), the hyperpolarizability ratio $\left( {R={\beta _{aac}}/{\beta _{ccc}}={\beta _{bbc}}/{\beta _{ccc}}} \right)$ is not exactly known and varying *R* in the range 0.1–0.5 yields tilt angles of 40°–0°, respectively. Based on the known perpendicular adsorption of CO on Pt, an exact *R*-value of 0.49 was determined. Polarization-dependent SFG spectra in the pressure range 10^−4^ to 36 mbar did not indicate any change of the tilt angle of adsorbed CO. Modeling also indicated a strong dependence of ${I_{{\text{ppp}}}}/{I_{{\text{ssp}}}}$ on the incidence angles of visible and IR laser beams. Complementing previous low temperature/low pressure data, room temperature CO adsorption on Pd(111) was examined from 10^−6^ to 250 mbar. The absolute PPP and SSP spectral intensities on Pt and Pd were simulated, as well as the expected ${I_{{\text{ppp}}}}/{I_{{\text{ssp}}}}$ ratios. Although CO on Pt and Pd should exhibit similar intensities (at high CO coverage), the higher ${I_{{\text{ppp}}}}/{I_{{\text{ssp}}}}$ ratio for Pd (48 vs. 27 on Pt) renders the detection of adsorbed CO in SSP spectra difficult. The presence or absence of CO species in SSP spectra can thus not simply be correlated to tilted or perpendicular CO molecules, respectively. Careful modeling, including not only molecular and interface properties, but also the experimental configuration (incidence angles), is certainly required even for seemingly simple adsorbate–substrate systems.

## Introduction

For many years, the surface science approach to heterogeneous catalysis was restricted to gas pressures of 10^−6^ mbar and below, giving rise to the well-known “pressure gap” problem [1–3]. This limitation was overcome when surface-sensitive methods (or modes thereof) were developed that could be operated at least in the mbar pressure range. Among the first were sum frequency generation (SFG) vibrational spectroscopy and high pressure scanning tunneling microscopy (HP-STM), both methods and their application to catalytic problems being pioneered by Somorjai and coworkers [4–6]. A specific asset of SFG was the ability to monitor adsorbed molecules in situ during the ongoing catalytic reaction at mbar gas pressure and elevated temperature, i.e. under technologically relevant conditions [7]. To date, Somorjai’s group has examined a vast range of reaction systems, mostly on Pt surfaces, spanning from ethylene, propylene and cyclohexene hydrogenation/dehydrogenation via CO oxidation/dissociation and CO/hydrocarbon coadsorption to selective aldehyde hydrogenation [8–13], to name just a few. As Pt model catalysts, various low-Miller-index as well as stepped and kinked single crystal surfaces were utilized (linking the SFG in situ work to Somorjai’s early high pressure cell studies without spectroscopy), complemented by thin films as supports for shape-controlled colloidal nanoparticles [14, 15]. This enabled to examine metal-support interactions for nanoparticles that often occur in reducing (hydrogen) atmosphere [16–18].

SFG vibrational spectroscopy can also be used to probe the orientation (tilt angle) of molecules adsorbed on metallic, semiconductor and insulator surfaces, making use of the dependence of intensities of different polarization combinations on the orientation of the surface molecule [19–23]. In this context, the molecular adsorbate structures of CO on Pt(111) [23, 24] and Pd(111) [22] have been studied, but results on the orientation (tilt angle) of CO on single-crystal surfaces were somewhat ambiguous. This is due to the complexity of the orientation analysis, which is based on polarization-dependent SFG spectroscopy, and the analysis/modeling of the observed intensities (which sometimes includes simplifications and/or assumptions that may not be justified). In this contribution, we revisit the benchmark systems of CO/Pt(111) and CO/Pd(111), employing a new SFG setup (UHV to mbar) [25] and specifically discuss the orientation analysis in detail, also reflecting analogies and differences to previous studies.

## Basic Theory of SFG

SFG is a second-order nonlinear optical process during which two photons of certain frequencies interact simultaneously with a surface molecule to instantaneously emit a new photon at the sum of the two frequencies. The unique advantage of SFG, distinct from other surface-sensitive techniques, is attributed to its interface selectivity. This originates from the fact that coherent second-order optical processes are forbidden in media with inversion symmetry, while they are allowed for an interface layer, where this centrosymmetry is naturally broken. The SFG intensity $\left( {{I_{SFG}}} \right)$ is proportional to the two incident laser intensities (${I_{Vis}}$ and ${I_{IR}}$) and the absolute square of second-order nonlinear susceptibility ($\chi _{{{\text{eff}}}}^{{{\text{(2)}}}}$), as shown in Eq. 1.

1 $${I_{SFG}} \propto {\left| {{\varvec{\upchi}}_{{{\text{eff}}}}^{{{\text{(2)}}}}} \right|^{\text{2}}}{I_{Vis}}{I_{IR}}$$

The surface susceptibility $\chi _{{{\text{eff}}}}^{{{\text{(2)}}}}$ is composed of a non-resonant ($\chi _{{NR}}^{{(2)}}$) and resonant part ($\chi _{R}^{{(2)}}$)

2 $$\chi _{{{\text{eff}}}}^{{{\text{(2)}}}}=\chi _{{NR}}^{{{\text{(2)}}}}+\chi _{R}^{{{\text{(2)}}}}={\chi _0}{e^{i\phi }}+\sum {\frac{{{\chi _q}}}{{{\omega _{IR}} - {\omega _q}+i{\Gamma _q}}}}$$

${\chi _0}$ is the magnitude of the non-resonant susceptibility $\chi _{{NR}}^{{(2)}}$ due to electronic excitations of the substrate and the adsorbate, and $\phi$ is its phase relative to the resonant term. ${\chi _q}$, ${\omega _q}$ and ${\Gamma _q}$ represent the resonance amplitude, frequency and damping constant of the *q*th vibrational mode, respectively. ${\omega _{IR}}$ is the frequency of the IR laser beam. In general, $\chi _{{NR}}^{{(2)}}$ should be small and real when the substrate is not resonant with either ${\omega _i}$ of incident visible, IR and output SFG beams. For dielectric interfaces, it is negligible. However, for metal or semiconductor substrate interfaces, $\chi _{{NR}}^{{(2)}}$ generally becomes complex and can no longer be ignored. $\chi _{{{\text{eff}}}}^{{{\text{(2)}}}}$ depends on the experimental polarization and geometry, and there are an infinite number of combinations of experimental configurations that can give different $\chi _{{{\text{eff}}}}^{{{\text{(2)}}}}$. In this paper, we mainly study $\chi _{{{\text{eff}}}}^{{{\text{(2)}}}}$ with a linear combination of independent experimental polarization combinations, namely, SSP (S-polarized sum frequency, S-polarized visible and P-polarized infrared) and PPP as shown in the following:

3 $$\begin{gathered} \chi _{{{\text{eff}},SSP}}^{{{\text{(2)}}}}={L_{yy}}\left( {{\omega _{SFG}}} \right){L_{yy}}\left( {{\omega _{Vis}}} \right){L_{zz}}\left( {{\omega _{IR}}} \right)\sin {\alpha _{IR}}{\chi _{yyz}} \\ \chi _{{{\text{eff}},PPP}}^{{{\text{(2)}}}}= - {L_{xx}}\left( {{\omega _{SFG}}} \right){L_{xx}}\left( {{\omega _{Vis}}} \right){L_{zz}}\left( {{\omega _{IR}}} \right)\cos {\alpha _{SFG}}\cos {\alpha _{Vis}}\sin {\alpha _{IR}}{\chi _{xxz}} \\ \;\; - {L_{xx}}\left( {{\omega _{SFG}}} \right){L_{zz}}\left( {{\omega _{Vis}}} \right){L_{xx}}\left( {{\omega _{IR}}} \right)\cos {\alpha _{SFG}}\sin {\alpha _{Vis}}\cos {\alpha _{IR}}{\chi _{xzx}} \\ \;\;+{L_{zz}}\left( {{\omega _{SFG}}} \right){L_{xx}}\left( {{\omega _{Vis}}} \right){L_{xx}}\left( {{\omega _{IR}}} \right)\sin {\alpha _{SFG}}\cos {\alpha _{Vis}}\cos {\alpha _{IR}}{\chi _{zxx}} \\ \;\;+{L_{zz}}\left( {{\omega _{SFG}}} \right){L_{zz}}\left( {{\omega _{Vis}}} \right){L_{zz}}\left( {{\omega _{IR}}} \right)\sin {\alpha _{SFG}}\sin {\alpha _{Vis}}\sin {\alpha _{IR}}{\chi _{zzz}} \\ \end{gathered}$$

Here ${\omega _{SFG}}$, ${\omega _{Vis}}$ and ${\omega _{IR}}$ are the frequencies; ${\alpha _{SFG}}$, ${\alpha _{Vis}}$ and ${\alpha _{IR}}$ are the angles (with respect to the surface normal), of the SFG signal, visible and IR laser beams, respectively. ${L_{ii}}({\omega _i})$ denotes the Fresnel factor at frequency ${\omega _i}$ for the local field corrections, which can be calculated with the knowledge of ${\alpha _i}$ and refractive indices *n*_1_ (medium 1, the air in which incident and SFG photons propagate), *n*_2_ (medium 2, the single crystal phase) and *n*′ (interfacial layer). In this paper, the *n*′ values were estimated by the modified Lorentz model, and the expression is $n^{\prime}=n{}_{1}{n_2}\sqrt {\frac{{6+{n_2}^{2} - {n_1}^{2}}}{{4{n_2}^{2}+2{n_1}^{2}}}}$ [26] ; when *n*_1_ = 1, then $n^{\prime}={n_2}\sqrt {\frac{{{n_2}^{2}+5}}{{4{n_2}^{2}+2}}}$ [27]. $\chi _{{ijk}}^{{(2)}}$ is the macroscopic sum frequency susceptibility, which is related to the microscopic hyperpolarizability tensor elements $\beta _{{i^{\prime}\,j^{\prime}\,k^{\prime}}}^{{(2)}}$ in the molecular coordinates system. $\chi _{{ijk}}^{{(2)}}$ can be deduced from SFG measurement with three different input/output polarization combinations, for example, SSP, SPS, PSS and PPP.

For ${C_{\infty v}}$ symmetry group, such as CO, OH, –CH, there are only two independent nonvanishing components in $\beta _{{i^{\prime}\,j^{\prime}\,k^{\prime}}}^{{(2)}}$, ${\beta _{ccc}}$ and ${\beta _{aac}}={\beta _{bbc}}$. Then, the non-zero macroscopic elements of $\chi _{{ijk}}^{{(2)}}$ for a rotationally isotropic interface which are obtained through integration over the Euler angles ($\phi$, azimuth angle; $\psi$, twist angle) can be expressed as follows [27, 28].

4 $$\begin{gathered} \chi _{{xxz}}^{{(2)}}=\chi _{{yyz}}^{{(2)}}=\frac{1}{2}{N_s}{\beta _{ccc}}\left[ {(1+R)\left\langle {\cos \theta } \right\rangle - (1 - R){{\left\langle {\cos \theta } \right\rangle }^3}} \right] \hfill \\ \chi _{{xzx}}^{{(2)}}=\chi _{{zxx}}^{{(2)}}=\chi _{{yzy}}^{{(2)}}=\chi _{{zyy}}^{{(2)}}=\frac{1}{2}{N_s}{\beta _{ccc}}\left[ {(1 - R)\left\langle {\cos \theta } \right\rangle - (1 - R){{\left\langle {\cos \theta } \right\rangle }^3}} \right] \hfill \\ \chi _{{zzz}}^{{(2)}}={N_s}{\beta _{ccc}}\left[ {R\left\langle {\cos \theta } \right\rangle +(1 - R){{\left\langle {\cos \theta } \right\rangle }^3}} \right] \hfill \\ \end{gathered}$$

Here $\beta _{{i^{\prime}\,j^{\prime}\,k^{\prime}}}^{{(2)}}$ is the molecular hyperpolarizability tensor. The hyperpolarizability ratio is $R={\beta _{aac}}/{\beta _{ccc}}={\beta _{bbc}}/{\beta _{ccc}}$. For a single bond with ${C_{\infty v}}$ symmetry, the *R*-value equals to the bond polarizability derivative ratio *r*. Different *R*-values will lead to different results in the SFG orientational analysis [21, 27, 28]. $\theta$ is the orientation angle of the moiety of the symmetry axis with respect to the surface normal. ${N_s}$ is the *effective* surface number density of molecules contributing to the SF signal. Then, based on Eqs. 1–4, we can determine the orientation ($\theta$) and/or molecular hyperpolarizability ratio (*R*) of the moiety by the measurements of the ratio of independent nonvanishing $\chi _{{ijk}}^{{(2)}}$ components assuming a *δ*-function distribution for $\theta$. The latter assumption is frequently applied because only a small angular distribution is expected.

## Experimental Section

The experiments on Pt(111) and Pd(111) single crystals were performed in a new UHV surface analysis system equipped with an SFG-compatible UHV-high pressure cell [25] (setup similar to that described in Refs. [2, 29]). Both Pt and Pd surfaces were cleaned by cycles of Ar ion bombardment (beam energy l.3 keV at 5 × 10^−6^ mbar of Ar, 10 min) and subsequent annealing at 1200 K for 2 min. If necessary, oxidation in 1 × 10^−6^ mbar O_2_ was used to remove carbon contamination. The surface structure was examined by LEED. Before transferring to the SFG cell, the clean single-crystal surface was exposed to 5 × 10^−7^ mbar of CO gas at room temperature via a leak valve, in order to passivate the surface and to avoid unwanted adsorption of impurities on the surface during sample transfer.

SFG was performed using a Nd:YAG (neodymium-doped yttrium aluminum garnet; Nd:Y_3_Al_5_O_12_) fundamental radiation of a PL2241 laser (EKSPLA, 1064 nm, 30 mJ/pulse), with a 20 ps pulse width and 50 Hz repetition rate. Part of the fundamental laser is frequency converted to second harmonics (532 nm) realized in K*DP nonlinear crystals. One part of laser pulses at 532 nm is used as visible input for the surface SFG experiment. Another part of the 532 nm beam is used to pump an optical parametric (BBO crystal)/difference frequency generation (AgGaS_2_ crystal) system and generate tunable infrared light from 1000–4300 cm^−1^ (2.3–10 µm) with a spectral width < 6 cm^−1^. The visible and infrared beams are spatially and temporally overlapped on the single-crystal surface in a co-propagating geometry at an incidence angle of 58.5° and 55° with respect to the surface normal, respectively. The energy is ~ 40 µJ/pulse for the visible beam and energies between 90 and 130 µJ/pulse were used for the tunable infrared pulse with frequencies between 1800 and 2160 cm^−1^. CaF_2_ and quartz windows served as entrance and exit ports for the incident laser beams and output SFG radiation, respectively. The SFG signal is filtered with a monochromator and detected with a photo-multiplier tube (PMT). The polarization of the visible light and SFG signal were switched between P and S using a Glan–Taylor prism and a half-wave plate, while the infrared polarization was always kept as P (an S-polarized field would be canceled on a metal surface) [30] .

## Results and Discussion

### SFG Spectra of CO on Pt(111)

**Fig. 1:**
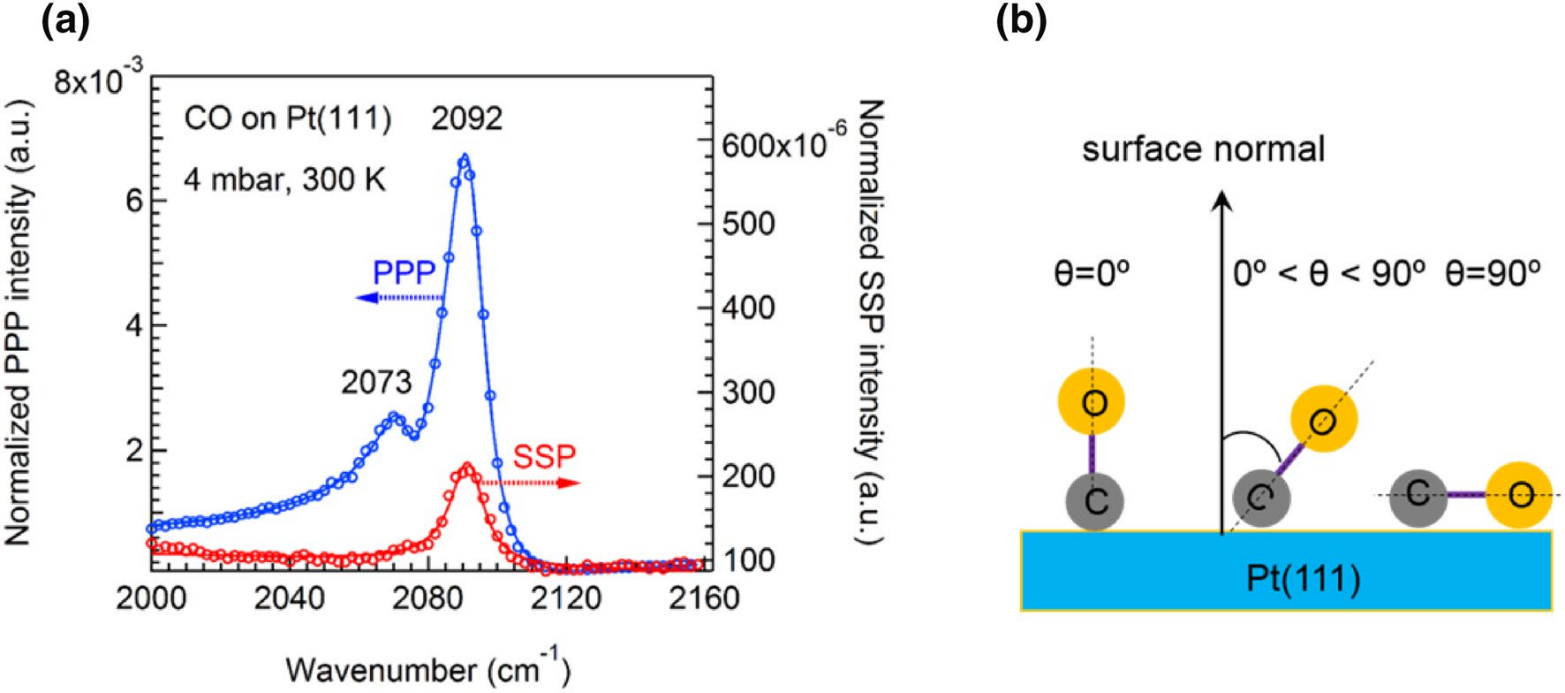
**a** Polarization-dependent SFG spectra of CO adsorption on Pt(111) at 4 mbar and 300 K. Symbols refer to experimental data. Blue circles: PPP polarization; Red circles: SSP polarization; Solid lines represent the global fitting curves with Lorentzian lineshapes (Eq. 2). **b** Schematic diagram of tilt angles of CO on a Pt single crystal surface

Polarization-dependent SFG spectra of CO/Pt(111) in the C–O stretch region were acquired at mbar pressure and 300 K. SSP and PPP spectra are presented in Fig. 1a. Because of the SFG selection rules [28], the SSP spectral intensity is much weaker than PPP. However, the spectra still had a good signal-to-noise ratio. The PPP spectrum of CO exhibits two peaks both attributed to linearly bonded CO molecules (with the carbon atom bound to a single Pt atom), but adsorbed at different sites of the single crystal. The sharp peak around 2092 cm^−1^ characterizes the on-top CO species adsorbed on terrace sites [31, 32], whereas the smaller shoulder around 2073 cm^−1^ is assigned to species adsorbed on step-sites on the surface [24, 33]. Unlike the PPP spectrum, in the SSP spectrum only on-top CO molecules adsorbed on terrace sites were detected, while the step-sites CO was not clearly observed (likely due to lower surface number density). The asymmetric lineshape of the SFG spectra, which is particularly pronounced for the lower frequency band in the PPP spectrum, is due to the interference of resonant and non-resonant terms (Eq. 2); particularly when ${\chi _{NR}}$ is real and has the same sign as ${\chi _q}$ [34].

The correspondence between C–O stretch frequencies and defect sites (steps or kinks) has been well documented for CO on Pt(111) using infrared spectroscopy [33, 34–38]. Frequencies for CO on defect sites are in general between 2058 and 2078 cm^−1^, lower than the frequencies of the terrace site vibrations. In a room-temperature SFG study of CO adsorbed on a disordered Pt(111) surface at a low coverage, a single vibrational frequency of 2072 cm^−1^ was reported by Daum and co-workers [24], but this frequency blue-shifted to 2093 cm^−1^ at higher coverage. For CO on a flat and well-ordered Pt(111) surface, the frequency of the C–O stretching vibration increases with exposure from 2083 to 2093 cm^−1^ at 300 K [24]. In addition, CO molecules prefer to adsorb on the step sites first, and then on the terrace-site of a defect-rich Pt(111) surface [33]. Similarly, studies of other surfaces with a high step density, such as Pt(533), also indicated that the adsorption of CO initially occurs in a linearly bonded configuration at step sites [37]. Thus, the appearance of a peak at 2073 cm^−1^ in Fig. 1a is attributed to the existence of steps on Pt(111).

Adsorbates on metal surfaces are usually considered not to produce (S or P) SFG signals for the SP (S-vis, P-IR) combination if the adsorbates have vibrational modes oscillating only along the surface normal [39]. The perpendicularly adsorbed CO on Pt(111) should thus not yield an SSP signal. However, in agreement with previous studies [22, 23] (in which some of us have demonstrated that this simplification is not valid), our experiment showed that both SSP and PPP spectra of on-top CO at terrace sites of the Pt(111) single crystal had a good signal-to-noise ratio. Fitting the peaks with Lorentzian lineshapes using Eq. 2 provided an accurate ${I_{{\text{ppp}}}}/{I_{{\text{ssp}}}}$ value of 27 for the on-top CO stretch mode. Next, we used the ratio of ${I_{{\text{ppp}}}}/{I_{{\text{ssp}}}}$ to analyze the orientation (tilt) angle (definition is illustrated in Fig. 1b) or molecular hyperpolarizability ratio (*R* = ${\beta _{aac}}/{\beta _{ccc}}={\beta _{bbc}}/{\beta _{ccc}}$) of on-top CO at the gas/Pt(111) interface.

#### Simulated ${I_{{\text{ppp}}}}/{I_{{\text{ssp}}}}$ Versus Tilt Angle (*θ*) and Hyperpolarizability Ratio (*R*-Value)

According to Eq. 4, the value alone is not sufficient to calculate the orientation angle of a certain surface molecule or functional group, because the hyperpolarizability ratio (*R*-value) must be known as well. Because SSP spectra of CO on single-crystal surfaces were rarely obtained, and the orientation angle of on-top CO was generally assumed to be 0° [22, 23], the orientation analysis has never been discussed in depth before. Almost two decades ago, only Badelli et al. [23] deduced an *R*-value of 0.6 and 1.5 for on-top CO and bridge-CO species on Pt(111) electrodes, respectively, based on the ratio of *I*_ssp_/*I*_ppp_ for CO molecules oriented parallel to the surface normal.

**Fig. 2:**
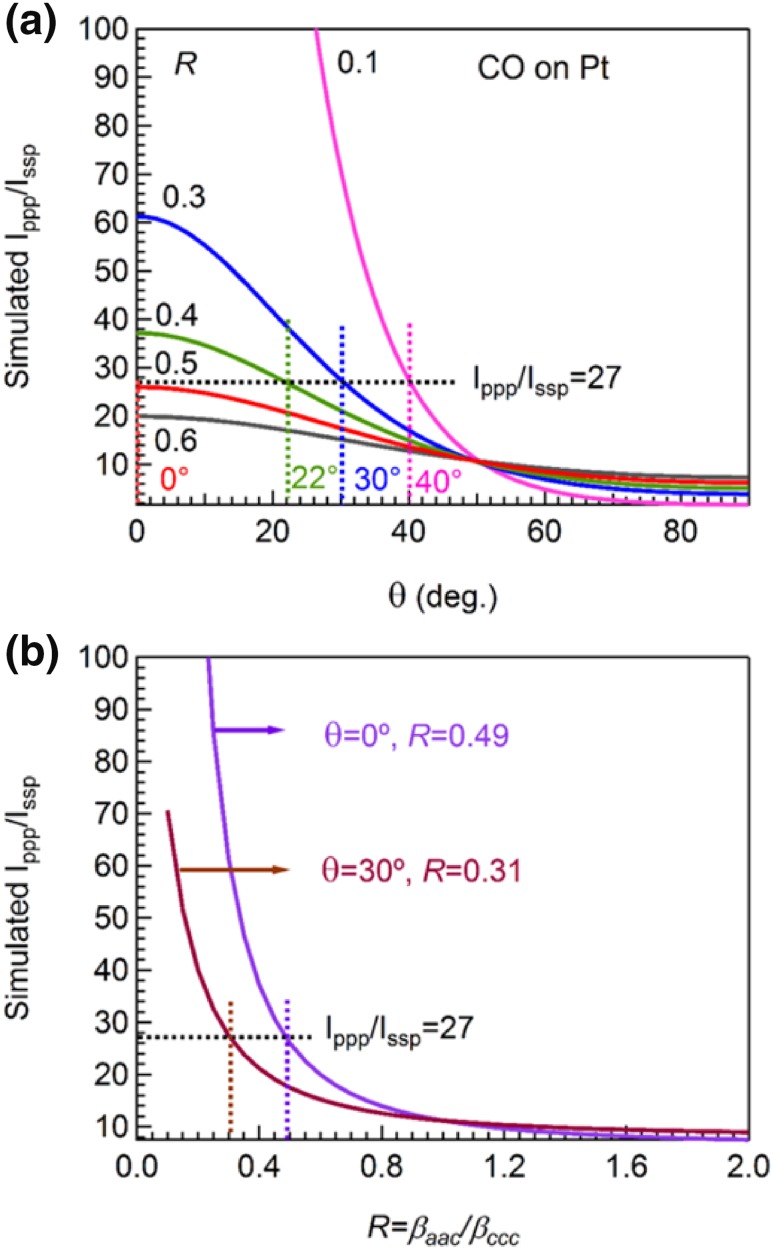
Simulated ${I_{{\text{ppp}}}}/{I_{{\text{ssp}}}}$ ratio as a function of **a** tilt angle (*θ*) for the CO molecule with a C_∞v_ symmetry and **b** molecular hyperpolarizability ratio (*R*). The following parameters were used in the simulation: ${\omega _{IR}}$ = 2090 cm^−1^, ${\alpha _{IR}}$ = 55°; ${\omega _{Vis}}$ = 532 nm, ${\alpha _{Vis}}$ = 58.5°; *N*_*s*_ = 1, *β*_ccc_ = 1. All refractive indices used for simulation are shown in Table 1

Since both the tilt angle θ and *R*-value are unknown parameters, we theoretically simulated the ratio of ${I_{{\text{ppp}}}}/{I_{{\text{ssp}}}}$ as a function of tilt angle with different *R*-values (Fig. 2a) and, vice versa, as a function of *R*-value assuming a 0° tilt angle (Fig. 2b). Refractive indices used in the simulations are summarized in Table 1. Incidence angles of visible (532 nm) and infrared (2092 cm^−1^) beams in the simulation were 58.5° and 55°, respectively.

**Table 1:** Bulk and interface refractive indices of air, Pt, and Pd at different wavelengths

	λ (µm)	n_1_ (air)	n_2_ (Pt) [40]	n′ (Pt)	n_2_ (Pd) [40]	n′ (Pd)
SFG	0.479	1	1.91 + 3.3i	1.14 + 1.4i	1.47 + 3.4i	0.89 + 1.4i
Vis	0.532	1	2.04 + 3.6i	1.18 + 1.6i	1.60 + 3.7i	0.94 + 1.6i
IR	4.785	1	3.89 + 18.9i	1.96 + 9.4i	3.3 + 20.2i	1.66 + 10.0i

For a CO molecule with a *C*_∞*v*_ symmetry, the *R*-value equals to its bond polarizability derivative ratio *r*. Generally, 0 ≤ *r* < 1 applies for a single chemical bond [28]. The ${I_{{\text{ppp}}}}/{I_{{\text{ssp}}}}$ value of 27, obtained from our SFG results, corresponds to R ≈ 0.5 (see Fig. 2a), assuming a 0° tilt angle, which is close to the 0.6 *R*-value reported earlier for on-top CO [23]. The difference of the calculated *R*-value may be due to different values of refractive indices of bulk and/or interfacial Pt (Ref. [23] did not specify them). However, the on-top CO molecules may not stand exactly upright on the Pt atoms. If we keep ${I_{{\text{ppp}}}}/{I_{{\text{ssp}}}}$= 27, the tilt angle of on-top CO is 40°, 30°, 22° and 0° in the case of adopting *R*-values of 0.1, 0.3, 0.4 and 0.5, respectively. When *R* = 0.6, there is no intersection at any tilt angle, according to our experimental result. Based on the discussion above, due to the uncertainty of *R*-values, it is difficult to determine the precise orientation of CO molecules on Pt surfaces without complementary information from other methods. For example, the Raman depolarization ratio (ρ) and bond polarizability derivative model have already been used to obtain a quantitative (but not fully accurate) description of the hyperpolarizability tensor ratios (*R*) in SFG analysis for stretching vibrational modes of the CH_3_, CH_2_ and CH groups [21, 41, 42]. The ρ-value can be accurately measured with polarized Raman techniques [28, 43, 44] and the *R*-value can be deduced from the ρ-value, for ${C_{\infty v}}$ symmetry, the relationship between *R* or *r*-value and *ρ*-value is $\rho=3\left/\left({4+5[(1+2r)/(1-r)]}^2\right)\right.$ [28]. Thus, if one can get the Raman depolarization ratio of CO molecules directly via experiments or theoretical calculations, the molecular hyperpolarizability or bond polarizability tensor ratio can be obtained, and the orientation analysis of adsorbed CO molecules becomes feasible. However, the ρ-value of CO molecules has not yet been reported.

Similarly, if the orientation angle is known, the precise *R*-value can also be determined. As the ratio of ${I_{{\text{ppp}}}}/{I_{{\text{ssp}}}}$ decreases, the *R*-value increases (Fig. 2b). More specifically, when ${I_{{\text{ppp}}}}/{I_{{\text{ssp}}}}$ > 40, the *R*-value is hardly sensitive to changes of ${I_{{\text{ppp}}}}/{I_{{\text{ssp}}}}$; whereas a small drop of the intensity ratio between 0 and 40 corresponds to a significant increase of the *R*-value. However, when the value of ${I_{{\text{ppp}}}}/{I_{{\text{ssp}}}}$ is smaller than 10, no reasonable *R*-value can be obtained anymore. Taking tilt angles of 0° and 30° as example, the exact *R*-value is 0.49 and 0.31 (Fig. 2b), respectively. Obviously, only 0.18 difference of *R*-value may lead to 30° tilt angle deviations, indicating that the determination of the orientation of surface CO molecules is rather difficult.

#### Pressure-Dependent SFG Spectra of CO Bond Stretching

**Fig. 3:**
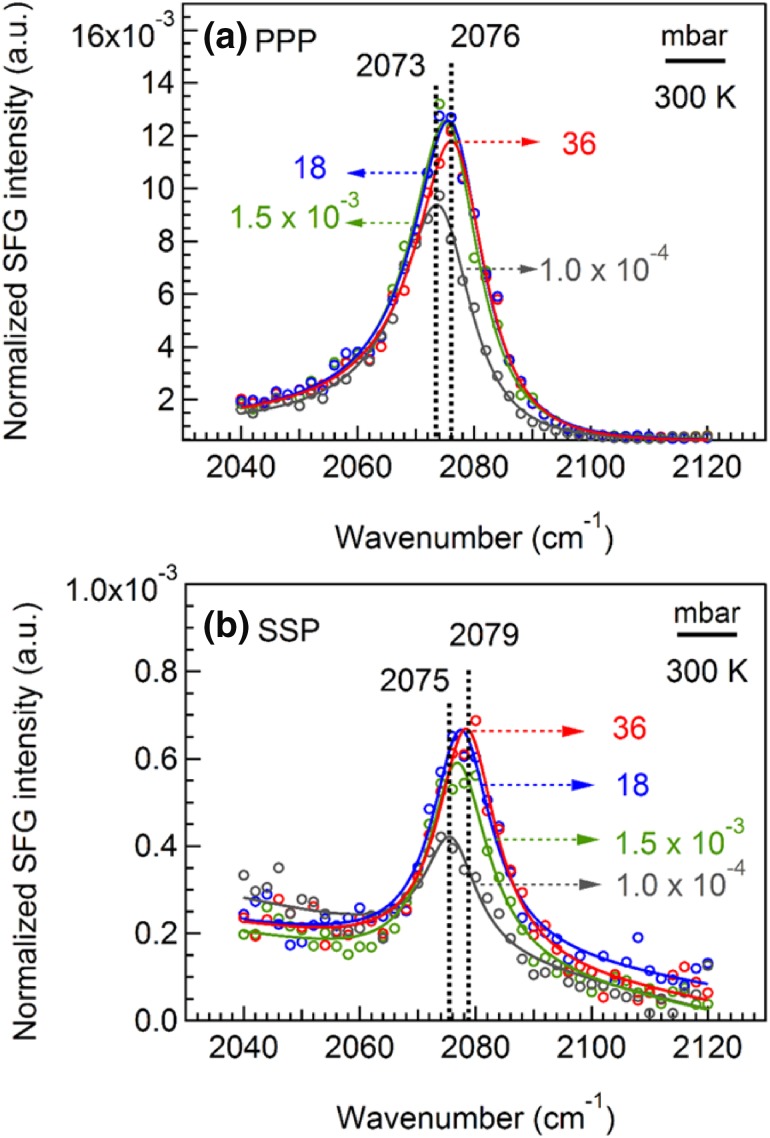
Pressure-dependent **a** PPP and **b** SSP spectra of CO on Pt(111) at 300 K

Taking advantage of the high pressure capability of SFG, pressure-dependent spectra of CO adsorption on Pt(111) were also measured (Fig. 3). The SSP and PPP spectral intensities simultaneously changed with the CO pressure, yielding almost the same ${I_{{\text{ppp}}}}/{I_{{\text{ssp}}}}$ ratio (20 ± 2) at different pressures. Apparently, the orientation of CO did not change at 300 K in the studied pressure range. Similarly, it was previously reported that there was no significant variation of the tilt angle upon increasing coverage of CO molecules adsorbed on (100) surfaces of body-centered cubic transition metals (Fe, Mo, Cr, and W) [45]. The peak position of CO at high pressure is shifted about 16 cm^−1^, as compared to those of Fig. 1. This can be explained by the CO-induced roughening of the surface, an effect that has also been reported by the Somorjai group [46]. CO-induced roughening was also observed for supported Pt nanoparticles [47].

There were only small changes in intensity and frequency upon increasing the pressure, since at 300 K saturation is almost reached at 10^−4^ mbar. This agrees with previous results of CO on smooth Pt(111) [31]. The observed increase of spectral intensity in PPP and SSP spectra can be attributed to the increasing surface coverage at constant orientation angle.

The absence of a vibrational band of bridge-bound CO on Pt(111) at 300 K can be explained by the strong broadening of the linewidth 2$\Gamma$ at high temperature [24]. Schweizer et al. [48], for example, had reported that CO on bridge sites had a linewidth of 37 cm^−1^ at 300 K. The linewidth broadening has been attributed to an order–disorder transition [49] with a partial loss of local order of the CO adlayer around 300 K [48].

#### The Effect of Incidence Angles of Visible and IR Laser Beams

**Fig. 4:**
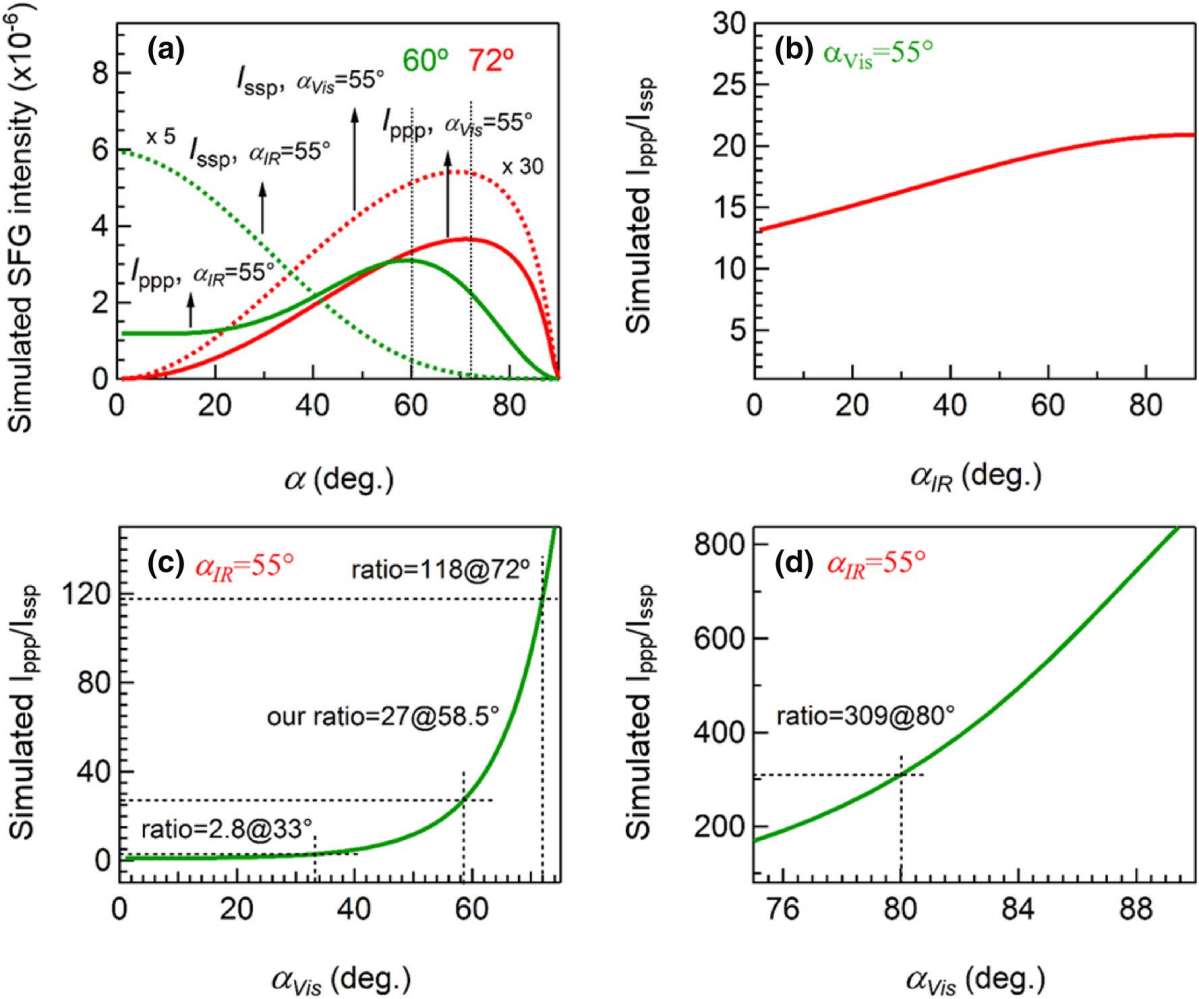
Simulations: **a** ${I_{{\text{ppp}}}}$ and ${I_{{\text{ssp}}}}$ versus incidence angles: fixed ${\alpha _{Vis}}$= 55°, ${I_{{\text{ppp}}}}$ (red solid line) and ${I_{{\text{ssp}}}}$ (red dashed line) versus ${\alpha _{IR}}$ and fixed ${\alpha _{IR}}$= 55°, ${I_{{\text{ppp}}}}$ (green solid line) and ${I_{{\text{ssp}}}}$ (green dashed line) versus ${\alpha _{Vis}}$. **b**${I_{{\text{ppp}}}}/{I_{{\text{ssp}}}}$ versus ${\alpha _{IR}}$ *(*fixed ${\alpha _{Vis}}$= 55°, red solid line). **c** ${I_{{\text{ppp}}}}/{I_{{\text{ssp}}}}$ versus ${\alpha _{Vis}}$ (fixed ${\alpha _{IR}}$= 55°, green solid line), ${\alpha _{Vis}}$= 0–75°. **d** ${I_{{\text{ppp}}}}/{I_{{\text{ssp}}}}$ versus ${\alpha _{Vis}}$ (fixed ${\alpha _{IR}}$= 55°, green solid line), ${\alpha _{Vis}}$= 75–90°. The following parameters were used in the simulation: ${\omega _{IR}}$ = 2090 cm^−1^, ${\omega _{Vis}}$ = 532 nm; *N*_*s*_ = 1, *β*_*ccc*_ = 1, θ = 0° and *R* = 0.49. All refractive indices used for simulation are shown in Table 1

SSP spectra of CO on Pt(111) have been rarely reported in previous SFG studies. However, as reported by Baldelli et al. [23] and herein, both SSP and PPP spectra with good signal-to-noise ratio can be acquired. In Ref. [23], a ${I_{{\text{ppp}}}}/{I_{{\text{ssp}}}}$ value of 1.25 (comparable spectral intensity of SSP and PPP) was obtained using incidence angles of 33° and 45° for visible and IR laser beams, respectively. As mentioned above, our results showed ${I_{{\text{ppp}}}}/{I_{{\text{ssp}}}}$ was 27 (spectral intensity of SSP is much smaller than PPP) for the experimental configuration of 55° and 58.5° for visible and IR laser beams, respectively. Obviously, the relative magnitude of the spectral intensity has a strong dependence on the incidence angles of the laser beams. In order to examine the effect of incidence angles on SFG spectra, we simulated ${I_{{\text{ppp}}}}$, ${I_{{\text{ssp}}}}$ (Fig. 4a) and ${I_{{\text{ppp}}}}/{I_{{\text{ssp}}}}$ of CO molecules on Pt (assuming θ = 0° and *R* = 0.49) for different incidence angles (${\alpha _{Vis}}$ and ${\alpha _{IR}}$). For better display, the curves of ${I_{{\text{ppp}}}}/{I_{{\text{ssp}}}}$ versus ${\alpha _{IR}}$ (Fig. 4b) and ${\alpha _{Vis}}$ are shown in separate diagrams, and ${I_{{\text{ppp}}}}/{I_{{\text{ssp}}}}$ versus ${\alpha _{Vis}}$ is even divided into two regions: one in the range of ${\alpha _{Vis}}$ = 0°–75° (Fig. 4c), and another in the range of ${\alpha _{Vis}}$ = 75°–90° (Fig. 4d). The parameters of refractive indices, used for the simulations, are shown in Table 1.

We first consider the spectral intensity changes. In Fig. 4a, for ${\alpha _{Vis}}$= 55°, ${I_{{\text{ppp}}}}$ and ${I_{{\text{ssp}}}}$ showed a strong but similar dependence on ${\alpha _{IR}}$, and reach the maximum for ${\alpha _{IR}}$= 72°. The ratio of ${I_{{\text{ppp}}}}/{I_{{\text{ssp}}}}$ is less sensitive to ${\alpha _{IR}}$ (Fig. 4b), as a result of the similar intensity response to ${\alpha _{IR}}$, which is consistent with a previous report [50]. However, for ${\alpha _{IR}}$= 55°, ${I_{{\text{ppp}}}}$ and ${I_{{\text{ssp}}}}$ depended on ${\alpha _{Vis}}$ differently, ${I_{{\text{ppp}}}}$ changed non-monotonically (increased first and then decreased), whereas ${I_{{\text{ssp}}}}$ decreased monotonically, resulting in a gradually increasing ${I_{{\text{ppp}}}}/{I_{{\text{ssp}}}}$ ratio (Fig. 4c, d).

In more detail, as evident from Fig. 4c, d, the value of ${I_{{\text{ppp}}}}/{I_{{\text{ssp}}}}$ has a very strong dependence on ${\alpha _{Vis}}$ at fixed ${\alpha _{IR}}$. A continuous increase of ${I_{{\text{ppp}}}}/{I_{{\text{ssp}}}}$ with increasing ${\alpha _{Vis}}$ indicates that the SSP signal will be rather difficult to obtain (as compared to PPP polarization), when a larger ${\alpha _{Vis}}$ is used. For example, when ${\alpha _{Vis}}$ is in the range of 0°–60°, ${I_{{\text{ppp}}}}/{I_{{\text{ssp}}}}$ is smaller than 30 (Fig. 4c), and ${I_{{\text{ppp}}}}$ reaches the maximum value at ${\alpha _{Vis}}$ = 60°; however, when ${\alpha _{Vis}}$ is larger than 70°, there are more than two orders of magnitude difference between ${I_{{\text{ppp}}}}$ and ${I_{{\text{ssp}}}}$ (Fig. 4d), e.g. when ${\alpha _{Vis}}$= 80°, ${I_{{\text{ppp}}}}$ is (unexpectedly) 309 times larger than ${I_{{\text{ssp}}}}$. Increasing ${\alpha _{Vis}}$ further, the ratio of ${I_{{\text{ppp}}}}/{I_{{\text{ssp}}}}$ could reach more than 800 (Fig. 4d), and ${I_{{\text{ppp}}}}$ decreases gradually (Fig. 4a, green solid line). This shows that especially ${\alpha _{Vis}}$ plays a crucial role in polarization-dependent SFG studies. A previous study of the effects of refractive index and ${\alpha _{Vis}}$ on the sum frequency generation intensity at air/liquid and solid/liquid interfaces also demonstrated the significance of ${\alpha _{Vis}}$ [51].

**Fig. 5:**
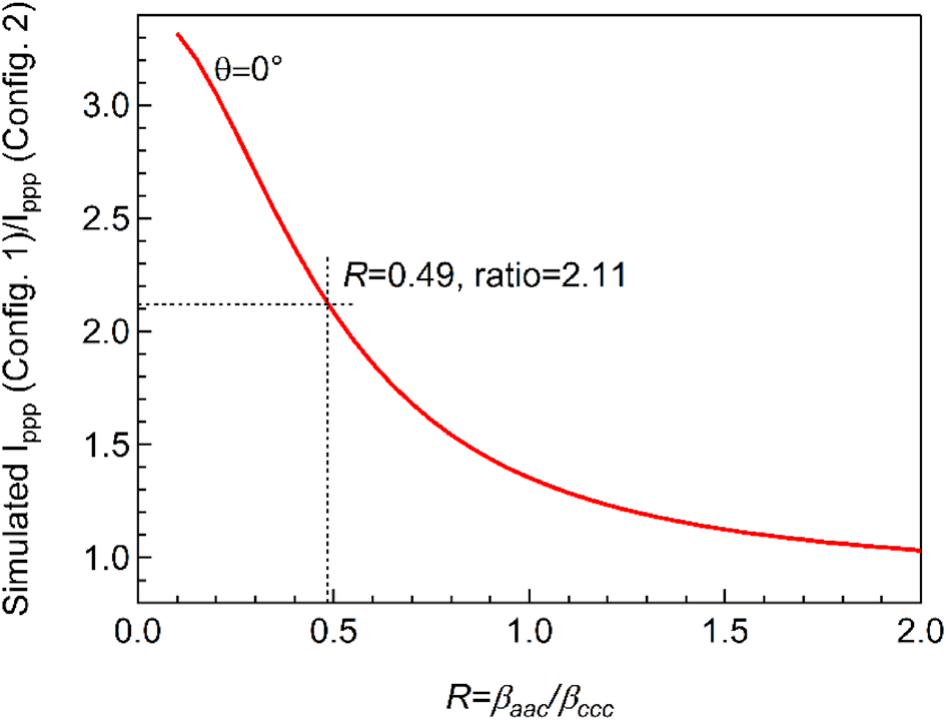
Simulated ratio of ${I_{{\text{ppp}}}}$ of Configuration 1 (${\alpha _{IR}}=55^\circ ,{\alpha _{Vis}}=58.5^\circ$) and ${I_{{\text{ppp}}}}$ of Configuration 2 (${\alpha _{IR}}=45^\circ ,{\alpha _{Vis}}=37^\circ$) as a function of molecular hyperpolarizability ratio (R) assuming $\theta =0^\circ$. The following parameters were used in the simulation: ${\omega _{IR}}$ = 2090 cm^−1^, ${\omega _{Vis}}$ = 532 nm; N_s_ = 1, β_ccc_ = 1. All refractive indices used for simulation are given in Table 1

In analogy to using the ratio of ${I_{{\text{ppp}}}}/{I_{{\text{ssp}}}}$ at a specific experimental configuration, the *R*-value at a selected tilt angle can also be deduced by measuring the ratio of ${I_{{\text{ppp}}}}$ collected from different experimental configurations. The two methods could then be used to mutually verify each other. Accordingly, for $R=0.49$ (deduced from ${I_{{\text{ppp}}}}/{I_{{\text{ssp}}}}=27$), the simulated ratio of ${I_{{\text{ppp}}}}$ of Configuration 1 (${\alpha _{IR}}=55^\circ ,{\alpha _{Vis}}=58.5^\circ$) and ${I_{{\text{ppp}}}}$ of Configuration 2 (${\alpha _{IR}}=45^\circ ,{\alpha _{Vis}}=37^\circ$) would be 2.11, as shown in Fig. 5. Unfortunately, due to limitations of our UHV-compatible SFG setup, the incidence angles of IR and visible beams can be only adjusted within a few degrees. We are thus currently unable to experimentally determine the ratio of I_ppp_ at different experimental configurations and to confirm the *R*-value that was deduced from I_ppp_/I_ssp_. However, the suggested method may be applied in the future using SFG setups that can widely vary the experimental configuration.

### SFG Spectra of CO on Pd(111)

Pd is used in many catalytic processes such as hydrogenation, oxidation/combustion, etc. [52, 53], and CO adsorption has also been used to characterize mono- and bimetallic surfaces [54, 55]. The pressure- and temperature-dependent CO adsorption on Pd(111) has thus been extensively studied, mostly using PPP polarization [56–59]. However, PPP and SSP spectra of CO/Pd(111) with good signal-to-noise ratio (${I_{{\text{ppp}}}}/{I_{{\text{ssp}}}}$= 10) were also obtained at 90 K, for an experimental configuration of ${\alpha _{Vis}}$= 54° and ${\alpha _{IR}}$= 56° [22] (similar to the current experimental conditions: ${\alpha _{Vis}}$=58.5°, ${\alpha _{IR}}$= 55°). At the low temperature (90 K), the 0.75 ML 2 × 2 saturation structure of CO was (predominantly) present and the signal of on-top CO (2108 cm^−1^), both in SSP and PPP spectra was much stronger than the signal of hollow (1890 cm^−1^) and bridge (1925 cm^−1^) bonded CO. At higher temperature (200 K), i.e. lower coverage, the signal of bridge (1951 cm^−1^) and on-top (2081 cm^−1^) CO species became comparable in PPP, whereas no adsorbate species could be detected in the SSP spectra.

**Fig. 6:**
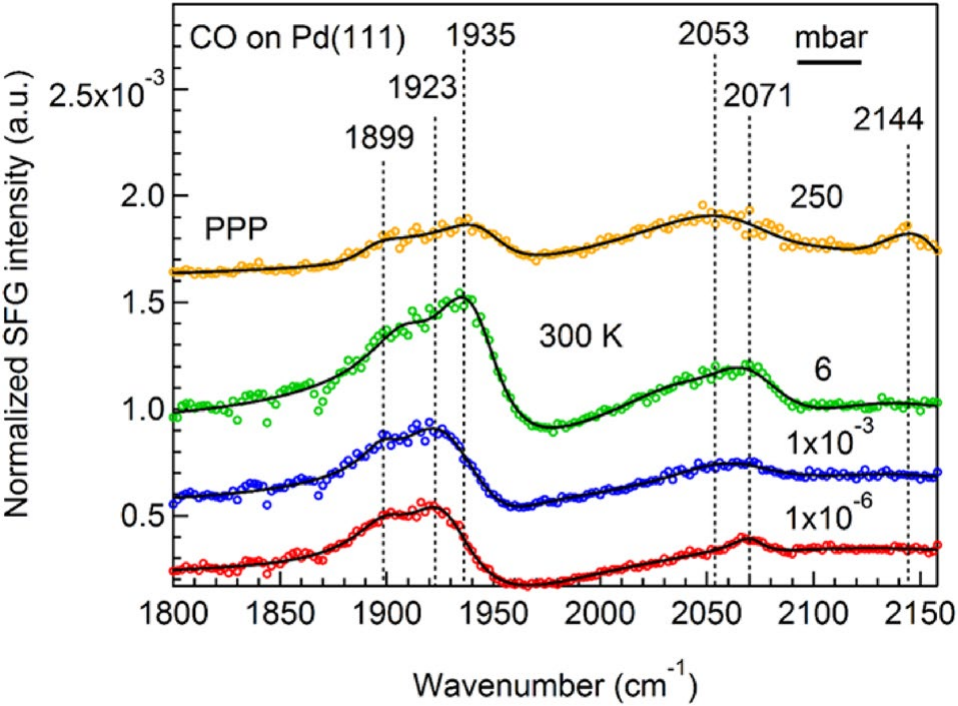
PPP spectra of CO adsorption on Pd(111) at 300 K

Herein, we have acquired PPP and SSP spectra for CO adsorption on Pd(111) at 300 K, complementing the measurements in Ref. [22], and have also used higher CO gas pressure (Fig. 6). Apparently, the hollow peak at ~ 1899 cm^−1^ (present as shoulder) and the bridge peak (1923 cm^−1^) rather than the on-top peak (2071 cm^−1^) dominated the PPP spectra [56–59] (due to coverage lower than that of the low-temperature PPP spectra in Ref. [22]). From 1 × 10^−6^ mbar to 250 mbar an approximate 12 cm^−1^ blue shift was observed for bridge-bonded CO due to increased coverage. For on-top CO a second peak appeared at 2053 cm^−1^ in 6 mbar. Apparently, at room temperature the CO overlayer is not perfectly ordered but rather a superposition of several CO structures in the coverage range 0.5–0.6 ML. For more detailed descriptions see Refs. [59, 60].

At 250 mbar, on-top CO not only existed in two species but there was also another higher frequency “feature” at 2144 cm^−1^ in PPP. Previous infrared reflection-adsorption spectra (IRAS) of 50 mbar CO on Pd(111) at 300 K also displayed a C–O vibrational feature, which is due to gas phase CO absorption (2143 cm^−1^) [61].

As shown in Fig. 6, the SFG spectral intensity gradually increased from low pressure to 6 mbar due to increasing surface CO coverage, but the spectral intensity strongly decreased at 250 mbar. As illustrated by Eq. 1, the SFG intensity depends on the effective surface IR and visible intensities. Although no SFG signal is generated by gas phase CO, the SFG process is indirectly influenced by the loss of IR-light via absorption by high-pressure CO gas. Thus, the (on-top) SFG signal may drop because the IR energy arriving at the sample is reduced in higher pressure CO. All SFG spectra were normalized by the IR and Vis energies but the energy-detector was located outside the input window, thus this normalization does not account for the CO gas phase absorption, which explains the unusual peak at 2144 cm^−1^ in PPP. It can be attributed to the 5.4 cm distance between the input window and the sample surface. In Refs. [56, 57, 59], using recessed IR windows, the distance was only 1.5 cm, so no IR absorption was observed even in 1000 mbar CO gas. For the current setup, normalization could be achieved by using GaAs references, by measuring the reflected IR light after the exit window, or by putting a 5.4 cm gas cell in front of the IR detector [2].

Unfortunately, in SSP polarization the peak intensities were too small for CO on Pd to be detected. In the following, we will explain the specific reason by comparing experimental and simulation results for CO on Pt and Pd.

### Comparison of CO on Pt(111) and Pd(111)

**Fig. 7:**
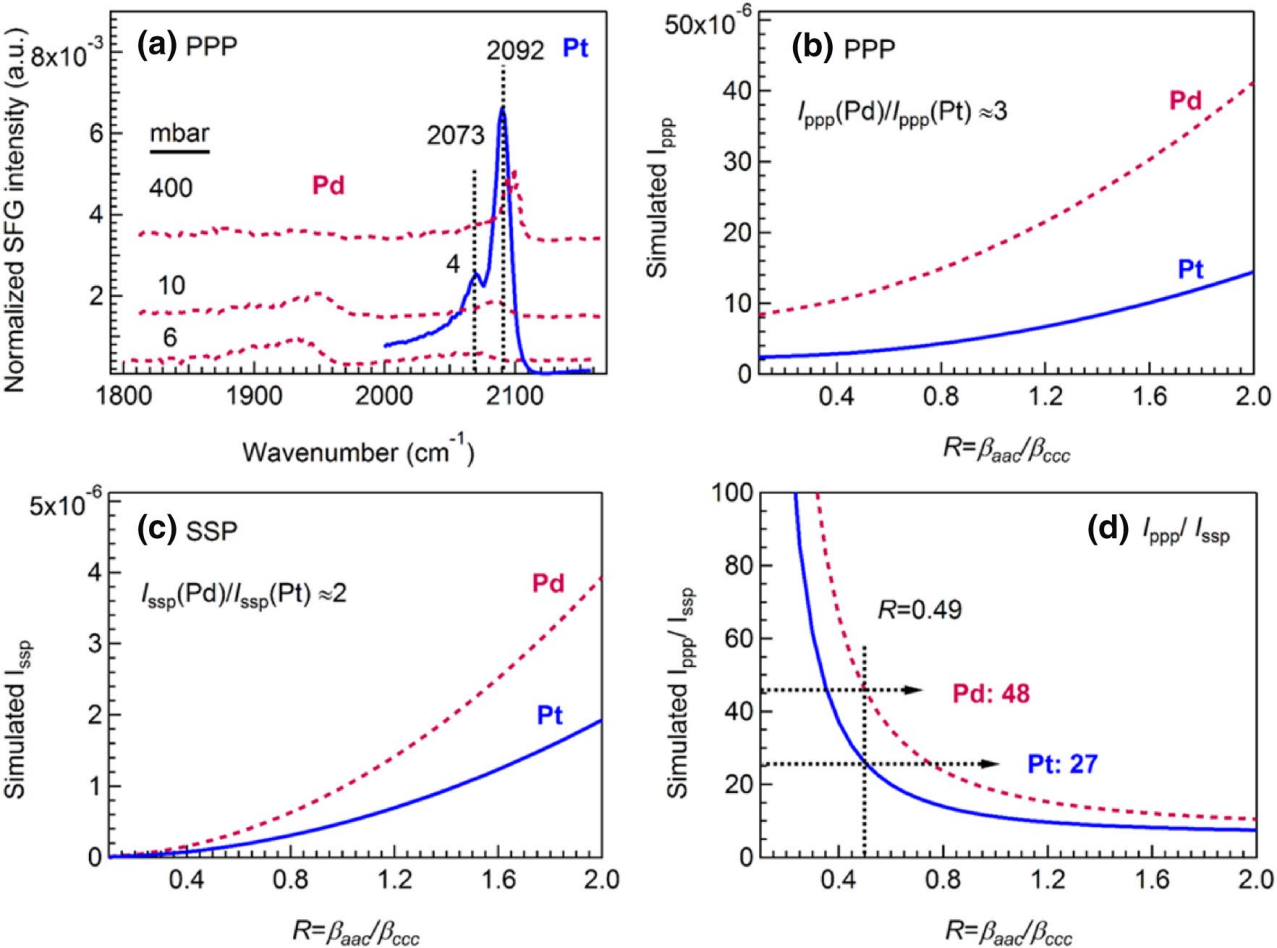
Comparison of spectral intensity of on-top CO on Pd(111) and Pt(111). **a** Experimental PPP spectra at 300 K, ${I_{{\text{ppp}}}}$(Pd) < ${I_{{\text{ppp}}}}$(Pt). Dark red: CO on Pd(111): 6 mbar (our data), 10 and 400 mbar (adapted from Refs. [56, 59]); blue: CO on Pt(111): 4 mbar. **b** Simulated ${I_{{\text{ppp}}}}$ versus *R*. **c** Simulated ${I_{{\text{ssp}}}}$ versus *R*. **d** ${I_{{\text{ppp}}}}/{I_{{\text{ssp}}}}$ versus *R*. The following parameters were used in the simulation: ${\omega _{IR}}$ = 2090 cm^−1^, ${\alpha _{IR}}$ = 55°; ${\omega _{Vis}}$ = 532 nm, ${\alpha _{Vis}}$ = 58.5°; *N*_*s*_ = 1, *β*_ccc_ = 1, and θ = 0°. All refractive indices used for simulation are shown in Table 1

In order to better illustrate the observed intensity differences, we have plotted the PPP spectra of CO on Pt(111) and Pd(111) together with some spectra from Refs [56, 59]. (Fig. 7a). Since on-top CO species were observed on both surfaces, we focus on disscussing SFG intensity changes of on-top CO. The PPP spectral intensity of CO on Pt(111) is obviously much larger than that on Pd(111). However, the on-top CO intensity on Pd(111) is strongly coverage dependent and we have thus included spectra at 10 and 400 mbar, when the higher CO coverage leads to intense CO peaks.

To better understand the distinctive intensity differences of the SFG experiments, we simulated ${I_{{\text{ppp}}}}$ (Fig. 7b), ${I_{{\text{ssp}}}}$ (Fig. 7c) and ${I_{{\text{ppp}}}}/{I_{{\text{ssp}}}}$ (Fig. 7d) versus *R* for on-top CO on Pt and Pd surfaces. For simulations, we assumed the same values of tilt angle *θ* = 0°, effective surface number density *N*_*s*_ = 1 and molecular hyperpolarizability *β*_*ccc*_ = 1 for Pt and Pd single crystals. Refractive indices used in the simulation can be found in Table 1. Interestingly, according to the theoretical simulation results, ${I_{{\text{ppp}}}}$ (Pd) is about three times larger than ${I_{{\text{ppp}}}}$ (Pt), and ${I_{{\text{ssp}}}}$ (Pd) is about two times larger than ${I_{{\text{ssp}}}}$ (Pt) in the whole range of *R* = 0.1–2. The simulated results are thus different from the experimental ones, for which Pt typically exhibits stronger SFG signal. Nevertheless, both experiment and theory deviate not too far in the order of magnitude of the SFG signal.

According to SFG theory, the factors influencing the SFG intensity of molecules adsorbing on different surfaces under the same experimental conditions (mainly referring to incidence angles of IR and visible beams), include the effective surface number density (*N*_s_), molecular orientation angles (*θ*), polarizations (e.g. SSP, PPP, etc.), Fresnel factors [*L*_ii_, related to laser incidence angles and refractive indices of bulk phases (n) and interfacial layer (n′)], molecular hyperpolarizability tensor (*β*_*ccc*_) and tensor ratio (*R* = *β*_*aac*_/*β*_*cc*c_ = *β*_*bbc*_/*β*_*ccc*_). All possible changes of the influence factors were considered for CO adsorption on Pt and Pd surfaces, except assuming identical *N*_s_. Thus, the conflicting results of experiments [${I_{{\text{ppp}}}}$(Pd) < ${I_{{\text{ppp}}}}$(Pt)] and simulations [${I_{{\text{ppp}}}}$(Pd) > ${I_{{\text{ppp}}}}$(Pt)], plus the fact that the intensity of an SFG peak is propotional to the square of *N*_s_, implies that the *N*_s_ of CO on the Pd surface should be much smaller than that on the Pt surface.

Assuming that the molecular hyperpolarizability ratio (*R*-value) of CO molecules is identical on different single crystal surfaces (based on the result of *R* = 0.49 and assumed *θ* = 0°) for CO on Pt, the theoretical value of ${I_{{\text{ppp}}}}$/${I_{{\text{ssp}}}}$ would be 48 for the Pd surface. This is much larger than the value of 27 for CO on Pt. Taking into account the small PPP signal on Pd (Fig. 7a) and the simulated large value of ${I_{{\text{ppp}}}}$/${I_{{\text{ssp}}}}$, this indicates that SSP spectra of on-top CO on Pd(111) are difficult to measure at room temperature. Consequently, no reasonable SSP spectra could be acquired.

## Conclusions

Polarization-dependent sum frequency generation (SFG) vibrational spectroscopy is a useful method, providing interface-specific spectra of adsorbed molecules, even at elevated gas pressure. For CO overlayers on Pt(111) and Pd(111) single crystal surfaces at room temperature, different polarization combinations (SSP and PPP) of the visible and SFG light were utilized to determine the molecular orientation (tilt angle) of CO. However, since the hyperpolarizability ratio $\left( {R={\beta _{aac}}/{\beta _{ccc}}={\beta _{bbc}}/{\beta _{ccc}}} \right)$ is not exactly known, we have rather determined this value (*R* = 0.49) by assuming perpendicular adsorption of CO (tilt angle of 0°). Pressure-dependent SFG spectra on Pt(111) (10^−4^ to 36 mbar) did not indicate any orientation change of adsorbed CO. Modeling the expected SFG ratio indicated a strong dependence on the experimental configuration, especially on the incidence angle of the visible beam.

Room temperature CO adsorption on Pd(111) was examined in the pressure range of 10^−6^ to 250 mbar but only PPP and no SSP spectra could be acquired. Modeling the absolute PPP and SSP spectral intensities on Pt and Pd, as well as the expected ratios, explained the absence of SSP signals on Pd. Care must apparently be taken even for the orientation analysis of seemingly simple adsorbate–substrate systems, with the interface model not only including refractive indices, but also the experimental configuration (incidence angles) and surface coverages.

## References

[1] Somorjai GA, Rupprechter G (1998). The flexible surface. J Chem Educ.

[2] Rupprechter G, Gates B, Knözinger H (2007). Sum frequency generation and polarization-modulation infrared reflection absorption spectroscopy of functioning model catalysts from ultrahigh vacuum to ambient pressure. Advances in catalysis.

[3] Rupprechter G, Wandelt K (2016). Surface science approach to heterogeneous catalysis. Surface and interface science: solid-gas interfaces I.

[4] Somorjai GA, McCrea KR, Gates B, Knözinger H (2000). Sum frequency generation: surface vibrational spectroscopy studies of catalytic reactions on metal single-crystal surfaces. Advances in catalysis.

[5] Somorjai GA, Rupprechter G (1999). Molecular studies of catalytic reactions on crystal surfaces at high pressures and high temperatures by infrared-visible sum frequency generation (SFG) surface vibrational spectroscopy. J Phys Chem B.

[6] Somorjai GA, Su XC, McCrea KR, Rider KB (1999). Molecular surface studies of adsorption and catalytic reaction on crystal (Pt, Rh) surfaces under high pressure conditions (atmospheres) using sum frequency generation (SFG)—surface vibrational spectroscopy and scanning tunneling microscopy (STM). Top Catal.

[7] Kung KY, Chen P, Wei F, Rupprechter G, Shen YR, Somorjai GA (2001). Ultrahigh vacuum high-pressure reaction system for 2-infrared 1-visible sum frequency generation studies. Rev Sci Instrum.

[8] Yang MC, Chou KC, Somorjai GA (2004). The structures and reactions of linear and cyclic C_6_ hydrocarbons adsorbed on the Pt(111) crystal surface by sum frequency generation vibrational spectroscopy pressure, temperature, and H_2_ coadsorption effects. J Phys Chem B.

[9] Yang M, Somorjai GA (2004). Unusual hydrogen effect in olefin dehydrogenation: 1-methylcyclohexene dehydrogenation initiated by excess hydrogen over Pt(111) surfaces, a combined sum frequency generation spectroscopy and kinetic study. J Phys Chem B.

[10] Yang M, Somorjai GA (2004). Adsorption and reactions of C_6_ hydrocarbons at high pressures on Pt(111) single-crystal surfaces studied by sum frequency generation vibrational spectroscopy: mechanisms of isomerization and dehydrocyclization of n-hexane. J Am Chem Soc.

[11] Thompson CM, Carl LM, Somorjai GA (2013). Sum frequency generation study of the interfacial layer in liquid-phase heterogeneously catalyzed oxidation of 2-propanol on platinum: effect of the concentrations of water and 2-propanol at the interface. J Phys Chem C.

[12] Sapi A, Liu FD, Cai XJ, Thompson CM, Wang HL, An KJ, Krier JM, Somorjai GA (2014). Comparing the catalytic oxidation of ethanol at the solid-gas and solid liquid interfaces over size-controlled Pt nanoparticles: striking differences in kinetics and mechanism. Nano Lett.

[13] Zhu ZW, Barroo C, Lichtenstein L, Eren B, Wu CH, Mao BH, de Bocarme TV, Liu Z, Kruse N, Salmeron M, Somorjai GA (2014). Influence of step geometry on the reconstruction of stepped platinum surfaces under coadsorption of ethylene and CO. J Phys Chem Lett.

[14] Baker LR, Kennedy G, Krier JM, Van Spronsen M, Onorato RM, Somorjai GA (2012). The role of an organic cap in nanoparticle catalysis: reversible restructuring of carbonaceous material controls catalytic activity of platinum nanoparticles for ethylene hydrogenation and methanol oxidation. Catal Lett.

[15] Krier JM, Michalak WD, Baker LR, An K, Komvopoulos K, Somorjai GA (2012). Sum frequency generation vibrational spectroscopy of colloidal platinum nanoparticle catalysts: disordering versus removal of organic capping. J Phys Chem C.

[16] Wang D, Penner S, Su DS, Rupprechter G, Hayek K, Schlögl R (2003). Silicide formation on a Pt/SiO_2_ model catalyst studied by TEM, EELS, and EDXS. J Catal.

[17] Penner S, Wang D, Su DS, Rupprechter G, Podloucky R, Schlögl R, Hayek K (2003). Platinum nanocrystals supported by silica, alumina and ceria: metal-support interaction due to high-temperature reduction in hydrogen. Surf Sci.

[18] Hayek K, Goller H, Penner S, Rupprechter G, Zimmermann C (2004). Regular alumina-supported nanoparticles of iridium, rhodium and platinum under hydrogen reduction: structure, morphology and activity in the neopentane conversion. Catal Lett.

[19] Liu WT, Zhang LN, Shen YR (2006). Interfacial structures of methanol: water mixtures at a hydrophobic interface probed by sum-frequency vibrational spectroscopy. J Chem Phys.

[20] Feng RR, Guo Y, Wang HF (2014). Reorientation of the “free OH” group in the top-most layer of air/water interface of sodium fluoride aqueous solution probed with sum-frequency generation vibrational spectroscopy. J Chem Phys.

[21] Li X, Deng GH, Feng RJ, Lin K, Zhang Z, Bai Y, Lu Z, Guo Y (2016). Salt effect on molecular orientation at air/liquid methanol interface. Chin Chem Lett.

[22] Galletto P, Unterhalt H, Rupprechter G (2003). The molecular orientation of CO on Pd(111): a polarization-dependent SFG study. Chem Phys Lett.

[23] Baldelli S, Markovic N, Ross P, Shen YR, Somorjai GA (1999). Sum frequency generation of CO on (111) and polycrystalline platinum electrode surfaces: evidence for SFG invisible surface CO. J Phys Chem B.

[24] Klünker C, Balden M, Lehwald S, Daum W (1996). CO stretching vibrations on Pt(111) and Pt(110) studied by sum-frequency generation. Surf Sci.

[25] Roiaz M, Pramhaas V, Li X, Rameshan C, Rupprechter G (2018). Atmospheric pressure reaction cell for operando sum frequency generation spectroscopy of ultrahigh vacuum grown model catalysts. Rev Sci Instr.

[26] Li X (2016) Investigation of molecular orientation and structure at air/electrolyte solution interface and phase transition of PNIPAM polymer at the air/H_2_O interface using sum frequency generation vibrational spectroscopy. PhD Thesis, Institute of Chemistry, University of Chinese Academy of Sciences, Peking

[27] Zhuang X, Miranda PB, Kim D, Shen YR (1999). Mapping molecular orientation and conformation at interfaces by surface nonlinear optics. Phys Rev B.

[28] Wang HF, Gan W, Lu R, Rao Y, Wu BH (2005). Quantitative spectral and orientational analysis in surface sum frequency generation vibrational spectroscopy (SFG-VS). Int Rev Phys Chem.

[29] Rupprechter G, Dellwig T, Unterhalt H, Freund HJ (2001). CO adsorption on Ni(100) and Pt(111) studied by infrared-visible sum frequency generation spectroscopy: design and application of an SFG-compatible UHV-high-pressure reaction cell. Top Catal.

[30] Hoffmann FM (1983). Infrared reflection-adsorption spectroscopy of adsorbed molecules. Surf Sci Rep.

[31] Rupprechter G, Dellwig T, Unterhalt H, Freund HJ (2001). High-pressure carbon monoxide adsorption on Pt(111) revisited: a sum frequency generation study. J Phys Chem B.

[32] Harle H, Mendel K, Metka U, Volpp HR, Willms L, Wolfrum J (1997). Temperature dependence (90–440 K) of the vibrational spectra of CO adsorbed on platinum(111) studied by sum-frequency generation. Chem Phys Lett.

[33] Agrawal VK, Trenary M (1991). An infrared study of NO adsorption at defect sites on Pt(111). Surf Sci.

[34] Miranda PB, Shen YR (1999). Liquid interfaces: a study by sum-frequency vibrational spectroscopy. J Phys Chem B.

[35] Reuttrobey JE, Doren DJ, Chabal YJ, Christman SB (1990). CO diffusion on Pt(111) with time-resolved infrared-pulsed molecular-beam methods—critical tests and analysis. J Chem Phys.

[36] Reuttrobey JE, Doren DJ, Chabal YJ, Christman SB (1988). Microscopic CO diffusion on a Pt(111) surface by time-resolved infrared-spectroscopy. Phys Rev Lett.

[37] Hayden BE, Kretzschmar K, Bradshaw AM, Greenler RG (1985). An infrared study of the adsorption of CO on a stepped platinum surface. Surf Sci.

[38] Greenler RG, Burch KD, Kretzschmar K, Klauser R, Bradshaw AM, Hayden BE (1985). Stepped single-crystal surfaces as models for small catalyst particles. Surf Sci.

[39] Yuzawa T, Shioda T, Kubota J, Onda K, Wada A, Domen K, Hirose C (1998). Polarization characteristics from SFG spectra of clean and regulatively oxidized Ni(100) surfaces adsorbed by propionate and formate. Surf Sci.

[40] Rakic AD, Djurisic AB, Elazar JM, Majewski ML (1998). Optical properties of metallic films for vertical-cavity optoelectronic devices. Appl Opt.

[41] Wei X, Hong SC, Zhuang XW, Goto T, Shen YR (2000). Nonlinear optical studies of liquid crystal alignment on a rubbed polyvinyl alcohol surface. Phys Rev E.

[42] Oh-e M, Yokoyama H, Baldelli S (2004). Structure of the glycerol liquid/vapor interface studied by sum-frequency vibrational spectroscopy. Appl Phys Lett.

[43] Colles MJ, Griffith.Je (1972). Relative and absolute raman scattering cross-sections in liquids. J Chem Phys.

[44] Li ZG, Wang JX, Li YM, Xiong W (2016). Solving the “magic angle” challenge in determining molecular orientation heterogeneity at interfaces. J Phys Chem C.

[45] Scheijen FJE, Ferre DC, Niemantsverdriet JW (2009). Adsorption and dissociation of CO on body-centered cubic transition metals and alloys: effect of coverage and scaling relations. J Phys Chem C.

[46] McCrea K, Parker JS, Chen PL, Somorjai G (2001). Surface structure sensitivity of high-pressure CO dissociation on Pt(557), Pt(100) and Pt(111) using sum frequency generation surface vibrational spectroscopy. Surf Sci.

[47] Haghofer A, Sonström P, Fenske D, Föttinger K, Schwarz S, Bernardi J, Al-Shamery K, Bäumer M, Rupprechter G (2010). Colloidally prepared Pt nanowires versus impregnated Pt nanoparticles: comparison of adsorption and reaction properties. Langmuir.

[48] Schweizer E, Persson BNJ, Tüshaus M, Hoge D, Bradshaw AM (1989). The potential-energy surface, vibrational phase relaxation and the order-disorder transition in the adsorption system Pt(111)-CO. Surf Sci.

[49] Ertl G, Neumann M, Streit KM (1977). Chemisorption of CO on Pt(111) surface. Surf Sci.

[50] Gan W, Wu BH, Chen H, Guo Y, Wang HF (2005). Accuracy and sensitivity of determining molecular orientation at interfaces using sum frequency generation vibrational spectroscopy. Chem Phys Lett.

[51] Li X, Feng RJ, Wang JJ, Zhang Z, Lu Z, Guo Y (2015). Role of refractive index in sum frequency generation intensity of salt solution interfaces. Chin Chem Lett.

[52] Demoulin O, Rupprechter G, Seunier I, Le Clef B, Navez M, Ruiz P (2005). Investigation of parameters influencing the activation of a Pd/gamma-alumina catalyst during methane combustion. J Phys Chem B.

[53] de la Fuente OR, Borasio M, Galletto P, Rupprechter G, Freund HJ (2004). The influence of surface defects on methanol decomposition on Pd(111) studied by XPS and PM-IRAS. Surf Sci.

[54] Dellwig T, Rupprechter G, Unterhalt H, Freund HJ (2000). Bridging the pressure and materials gaps: high pressure sum frequency generation study on supported Pd nanoparticles. Phys Rev Lett.

[55] Rameshan C, Weilach C, Stadlmayr W, Penner S, Lorenz H, Hävecker M, Blume R, Rocha T, Teschner D, Knop-Gericke A, Schlögl R, Zemlyanov D, Memmel N, Rupprechter G, Klötzer B (2010). Steam reforming of methanol on PdZn near-surface alloys on Pd(111) and Pd foil studied by in-situ XPS, LEIS and PM-IRAS. J Catal.

[56] Unterhalt H, Rupprechter G, Freund HJ (2002). Vibrational sum frequency spectroscopy on Pd(111) and supported Pd nanoparticles: CO adsorption from ultrahigh vacuum to atmospheric pressure. J Phys Chem B.

[57] Rupprechter G, Unterhalt H, Morkel M, Galletto P, Hu LJ, Freund HJ (2002). Sum frequency generation vibrational spectroscopy at solid-gas interfaces: CO adsorption on Pd model catalysts at ambient pressure. Surf Sci.

[58] Bourguignon B, Carrez S, Dragnea B, Dubost H (1998). Vibrational spectroscopy of imperfect CO/Pd(111) surfaces obtained by adsorption between 150 and 230 K. Surf Sci.

[59] Morkel M, Unterhalt H, Salmeron M, Rupprechter G, Freund HJ (2003). SFG spectroscopy from 10^– 8^ to 1000 mbar: less-ordered CO structures and coadsorption on Pd(111). Surf Sci.

[60] Rupprechter G, Unterhalt H, Morkel M, Galletto P, Dellwig T, Freund HJ (2003). Extending UHV studies to the mbar range: vibrational SFG spectroscopy of high-pressure CO adsorption on Pt(111) and Pd(111). Vacuum.

[61] Rupprechter G, Bandara A, Friedbacher G, Bubert H (2011). Sum frequency generation (SFG) spectroscopy. Surface and thin film analysis: a compendium of principles, instrumentation, and applications.

